# Outsourced eye care in the Finnish capital region: Switching from fee‐for‐service to bundled payment model

**DOI:** 10.1111/aos.17523

**Published:** 2025-05-19

**Authors:** Susanna Moisander, Jukka Ari Olavi Moilanen, Hans Henrik Konstantin Backström, Olga Marjasova, Marko Juhani Myöhänen, Taina Pauliina Nykänen, Mikko Juhani Pietilä, Paulus Torkki

**Affiliations:** ^1^ Department of Ophthalmology, Hospital Nova The wellbeing services county of Central Finland Jyväskylä Finland; ^2^ Department of Ophthalmology Helsinki University Hospital Helsinki Finland; ^3^ SOK Corporation Helsinki Finland; ^4^ EVLI Fund Management Company Helsinki Finland; ^5^ Elisa Oyj Helsinki Finland; ^6^ Mehiläinen Bulevardi Medical Clinic & Hospital Helsinki Finland; ^7^ Turku University Hospital, The welbeing services county of Southwest Finland Turku Finland; ^8^ Department of Public Health, Faculty of Medicine University of Helsinki Helsinki Finland

**Keywords:** bundled payment, fee‐for‐service, ophthalmology, outsourcing, reimbursement

## Abstract

**Purpose:**

The demand for specialised eye care has quadrupled over the last two decades, driven by an aging population and the advent of new treatments. This surge has led to increased outsourcing by the Helsinki and Uusimaa Hospital District (HUS) Eye Hospital, initially relying on a fee‐for‐service model. This model, however, has been marked by excessive bureaucracy and transaction costs. Between 2019 and 2021, HUS Eye Hospital embarked on a comparative analysis of outsourcing models to identify the most cost‐effective, efficient and suitable approach.

**Methods:**

After the review of literature and stakeholder interviews, three different outsourcing models were assessed: fee‐for‐service, capitation and bundled payment. The models were evaluated against each other and HUS insourcing using SWOT analysis, custom‐made evaluation matrices and cost analysis.

**Results:**

The evaluation identified bundled payments as the most promising model for HUS eye care outsourcing. Under this model, patients diagnosed with or suspected of having eye diseases are referred to external service providers for a predetermined period, encompassing follow‐up and treatment, with providers receiving a fixed payment for each assigned patient.

**Conclusion:**

Determining the optimal outsourcing model for HUS Eye Hospital was a complex process and necessitated the development of a new quality control system. The selection of bundled payment as the preferred method required an adaptation of it to the national requirements and legislation. While bundled payments have been successfully implemented in other medical specialties, their application in ophthalmology is novel, with forthcoming data from HUS Eye Hospital expected to shed further light on its effectiveness.

## 1 INTRODUCTION

In an era of tight budgets and heightened scrutiny on spending, the government's ability to extract more value from public‐sector outsourcing is crucial, yet not an easy task (Porter, 2010). It aims to utilise the opportunities in the supplier market to enable the needs of the customer to be fulfilled in the optimal way for the organisation. The most important drivers for outsourcing in healthcare units are (1) cost reduction, (2) risk mitigation, (3) adapting to quick changes without jeopardising internal resources and (4) value stream redefining (Guimarâes & de Carvalho, 2011). If outsourcing contracts and procurement processes are managed poorly, consequences can include cost overruns, financial problems with the private contractor and poor service quality. Six key success factors in the outsourcing procedure include tailoring the request for proposal process to identify key objectives, getting the requirements right, promoting competition from suitable suppliers, aligning objectives with incentives, ensuring data transparency and its effective utilisation, and retaining in‐house capabilities for contract monitoring and governance (Foote et al., 2018). As part of a transformation toward a more value‐based healthcare system, emphasis should be on long‐term collaboration between payers and providers (de Bakker et al., 2012). National, organisational and professional needs also set complex requirements for the outsourcing process (Berry et al., 2021).

Outsourcing of medical services can be considered a high‐risk outsourcing (Padovani & Young, 2006). Outsourcing clinical services can lead to the loss of control of the quality of care. It can also lead to inconsistencies in standards of care and declines in patient and employee satisfaction (Berry et al., 2021). The shortage of healthcare professionals and rising demand for medical services necessitates outsourcing. However, this also creates a competitive market for professionals, with markedly higher income opportunities. Hence, it is critical to determine the extent and nature of tasks suitable for outsourcing to prevent the loss of public‐sector employees due to heightened labour demand resulting from outsourcing. Furthermore, the increase in outsourcing results in increased administrative workload in the public hospitals, thus reducing the attractiveness of the public sector (Van Der Burgt et al., 2020).

Demographic change drives service demand in special healthcare, likewise in ophthalmology. During the last two decades, service demand in eye care has constantly increased globally (Bourne et al., 2021) as well as in European countries (MacEwen et al., 2019) including Finland. This is due to the visual demands of modern society, improved opportunities for care, and to the demographics of the world's population (Abdulhussein & Abdul Hussein, 2023). The modern society requiring excellent visual acuity increases the pressure for earlier treatment for eye diseases (Lewallen et al., 2014).

The biggest ophthalmic unit in Finland (HUS Eye Hospital) had used fee‐for‐service‐based outsourcing since 2005. The annually growing need for outsourcing led to annually increasing costs, administrative workload and demand for resources. This adverse development generated a need to evaluate the old outsourcing model and to compose a new one. This process is depicted in this article. The guidelines of the Helsinki Declaration were followed during the process and writing of this article.

## 2 METHODS

Finland has a population of 5.59 million, with the majority living in southern Finland, particularly in the capital region. The proportion of the elderly population is growing, driven by a decrease in births and longer life expectancy (Statistics Finland, n.d.). Aging is a major driver of service demand in healthcare, including eye care, as age is the most important risk factor for the four most common eye conditions, that is age‐related macular degeneration (AMD), glaucoma, diabetic retinopathy and cataract, ‘the Big Four’ (Tuulonen et al., 2016).

The Finnish healthcare system, primarily funded by taxes and mandated by the Constitution (Finland's Ministry of Justice 731/1999, 1999), ensures universal adequate coverage and equality in access to social, health and medical services. Alongside the public sector, private companies provide health services for self‐ or insurance‐paid patients and provide occupational healthcare. These private companies vary from small doctor‐owned to large corporate‐owned units. All private companies can participate in competitive tendering of outsourced health services.

Until 2022, municipalities were responsible for healthcare provision, but since 2023, government funds support the healthcare and social welfare system (Finland's Ministry of Justice 612/2021, 2021). The purpose of this law is to promote and maintain the wellbeing and health of the population, as well as to ensure equal, interoperable and cost‐effective social and healthcare services across the entire country. Specialised medical care transitioned from a diagnosis‐related group (DRG) model to a capitation model, with the country divided into 21 wellbeing services counties responsible for all health and social services. This legislation mandates that if a municipality, or nowadays a wellbeing service county, cannot provide a required service, it must either outsource via tender or provide a service voucher.

Detailed legislation governs public procurement to enhance the use of public funds, promote the making of high‐quality, innovative and sustainable procurement, and ensure equal opportunities for businesses and other organisations to offer goods, services and construction contracts in public procurement competitions (Finland's Ministry of Justice 1397/2016, 2016). Over the years, due to the obligation to organise adequate healthcare in an equitable manner, there has been a pressure for some services to be increasingly outsourced. This has not been an intentional goal, but rather, a result of practical reasons due to constant pressure to increase patient numbers, a shortage of healthcare professionals and the complexity of services required by an aging population. This development has continued over several decades, remaining unaffected by political changes, and is therefore likely to persist in the future.

The publicly funded healthcare consists of ca 490 health centres providing public primary healthcare. Specialised care is provided by five university hospitals (including local area hospitals) and 15 central hospitals. Helsinki University Hospital (HUS) being the biggest healthcare provider and responsible for organising specialised healthcare in the Uusimaa region.

In 2024, of the ca 490 primary healthcare centres, ca. 9% are totally or partly outsourced. In specialised care, typically only specific units (for example ophthalmology unit) can be outsourced. All university hospitals and central hospitals have ophthalmology units. Of these, none of the university hospital units is outsourced. However, eight of the 15 (53%) central hospitals units are. Of the five university ophthalmology units, three (60%) rely on outsourcing to supplement needed services, and of the seven non‐outsourced central hospital ophthalmology units, five (71%) utilise outsourcing for some patient groups.

**FIGURE 1:**
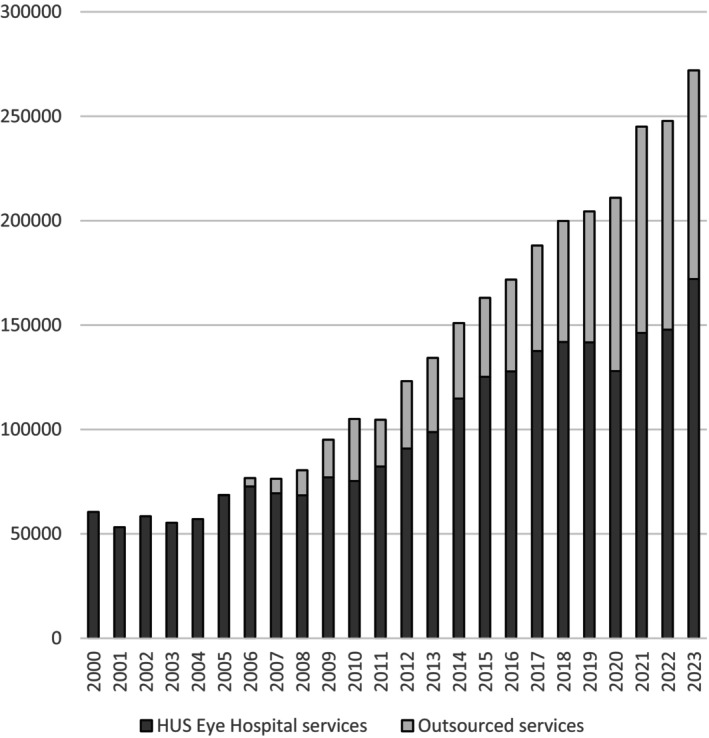
Annual outpatient visits, intravitreal injections and surgeries provided by HUS Eye Hospital and outsourced private providers.

HUS Eye Hospital, Finland's largest publicly funded academic eye hospital, provides specialised eye care in Helsinki and the surrounding areas, serving a population of 1.7 million, or 31% of Finland's total population. It also delivers university‐level eye care to the citizens of the HUS Catchment Area, which has a population of 2.2 million, representing 40% of Finland's population and offers centralised eye care services for the entire country. With annual operating costs of EUR 89.6 million in 2022 (HUS Year 2022. Online Annual Report, 2023), outsourcing has been a response to increasing demand since 2005. This was driven by the increasing demand for eye care, an aging population and inaccurate projections of eye care needs, which resulted in the consolidation of four specialised eye care units in the HUS area in the late 1990s. Despite an annual 5% increase in services provided by HUS Eye Hospital, outsourcing now comprises ca. 40% of all outpatient visits (Figure 1), with 58 000 outsourced consultations, totalling EUR 12.1 million in costs in 2021. These costs are part of the annual fixed budget, which cannot be exceeded.

Initially, only a small portion of outpatient visits and cataract operations were outsourced. However, increasing demand led to longer outsourcing contracts, which extended to two‐ to four‐year intervals by 2010. In 2011, cataract surgery was transitioned from outsourcing contracts to a service voucher system. The cataract voucher system has been effective, and its value has remained consistent over time. Consequently, cataract surgeries—representing 3–6 million euros of the total costs for outsourced services—were excluded from the reform.

By contrast, all outpatient outsourcing was structured around a fee‐for‐service model from the beginning, where individual visits were authorised by HUS and billed by private providers on a per‐visit basis. Over time, this led to a fragmented and complex network of contracts, which by 2016 had expanded to 49 options across eight categories. The public tendering process, conducted every 4 years, became increasingly cumbersome and time‐consuming. The rising volume of outsourced services also resulted in a significant administrative burden, requiring the equivalent of 3.5 full‐time ophthalmologists and 9.3 secretaries at HUS Eye Hospital in 2020.

**FIGURE 2:**
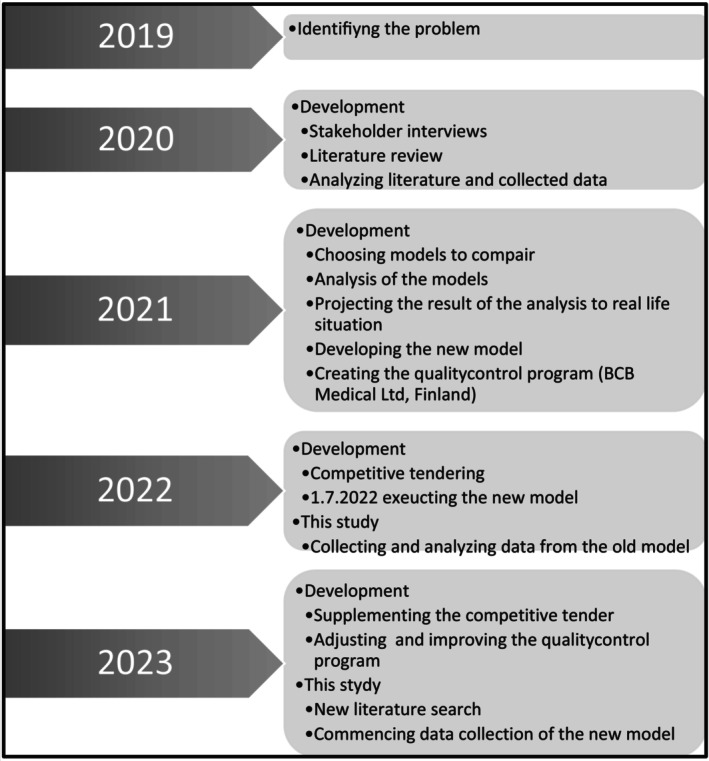
Flowchart of the developments of the choosing process and apposition of this study.

The increasing demand, bureaucracy and costs of outsourcing prompted HUS Eye Hospital to seek a streamlined process and a cost‐effective outsourcing model that maintains quality care for standard‐level ophthalmologic patients without creating a competing labour market for ophthalmologists. The initial planning of a new method for outsourcing was performed as an executive education (eMBA = executive Master of Business and administration) project in 2019–2021, led by the head of the department of HUS Eye Hospital, Jukka Moilanen. The group consisted of three medical doctors (in cardiology, gastrointestinal surgery and ophthalmology), an economist, an investment manager and an engineer. The criteria for a successful outsourcing model were established by evaluating various models and developing one tailored to HUS Eye Hospital's needs. Additionally, an implementation roadmap was devised. The timeline and phases of this process are illustrated in Figure 2, alongside the timeline for the current study and this article. Simultaneously, a new quality control registry was established with BCB Medical Ltd., Finland, for future monitoring. This registry became necessary due to the limitations of existing hospital information systems in providing insight into the quality and costs of outsourced services.

**FIGURE 3:**
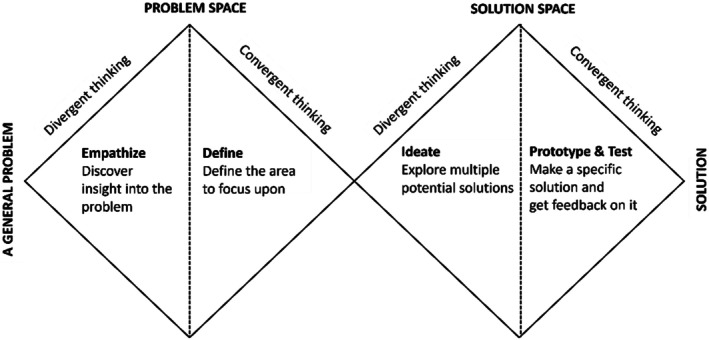
Double Diamond Model, modified from the original model of British Design Council.

The evaluation process utilised a combination of literature review, SWOT analyses, custom evaluation matrices and cost analysis to determine the best outsourcing approach for HUS Eye Hospital. It employed a mainly qualitative research approach, employing inductive reasoning to identify essential questions, explanations and relationships based on data (Williams, 2007). Due to the project's timeframe and data limitations, real‐life piloting was not feasible. The Double Diamond Model by the British Design Council (2003) served as the methodological approach (Figure 3) (Design Council, n.d.).

The possibility of insourcing was also reviewed, from a hypothetical cost comparison aspect. In it, the HUS Eye Hospital arranges all its eye care independently without outsourcing. In reality, despite the annual growth of patient volume in HUS Eye Hospital, the growth of outsourced services has been even faster in previous years (Figure 1).

### 2.1 Data collection from HUS Eye Hospital and stakeholder interviews

The work started by collecting and analysing data from HUS Eye Hospital outsourcing from 2005, when outsourcing started, to 2020, when the evaluation process was ongoing. Its records and documents provided information on the operating model of the hospital and past tenders. The previous outsourcing prices and costs were evaluated, and the volumes of patients treated both in HUS Eye Hospital and private providers were collected. No clinical treatment or quality data were available from the previous outsourcing model.

Patient experience and needs were assessed through analysis of key performance indicators (KPIs), Net Promoter Scores (NPS) from patients at HUS Eye Hospital and private providers. However, data collected by service providers were scarce and coincidental. Gathered patient feedback and interviews with HUS professionals also gave insight on quality assurance and management issues. Patient complaints from 2016 to 2020 were analysed. There were 195 complaints of which 41 (21%) involved outsourcing. The 128 Patient insurance centre decisions from 2016 to 2020 also were reviewed, of which 26 (20%) resulted in compensation payments to patients. None of these decisions leading to compensations involved outsourcing.

Structured face‐to‐face stakeholder interviews were conducted between August and September 2020 to ensure the crucial involvement of stakeholders in the project. Stakeholders, defined as individuals who can influence or are affected by the HUS Eye Hospital's objectives, were engaged to explore their challenges, motivations and needs. The objective was to gather insights into the current outsourcing model and to clarify expectations and ideal solutions for a new model. Data collection began with interviews of five HUS employees, followed by interviews with seven representatives from three major private providers involved in the previous outsourcing model (Table 1). All interviews were conducted by the six project team members, with the exception of interviewee #1, a project team member who was interviewed by the other team members. Each interviewee had been actively involved in the organisation of the ongoing outsourcing services. They were asked to identify the major issues with the current outsourcing model, suggest potential changes and describe their ideal model. The responses received guided the subsequent discussion, which lasted between 60 and 90 min.

**TABLE 1:** Stakeholder interviews.

Interview	Stakeholder	Date
#1	Representative from HUS Eye Hospital management, head of HUS Eye Hospital	19/08/2020
#2	Two HUS Eye Hospital ophthalmologists involved in managing outsourcing	19/08/2020
#3	HUS Eye Hospital ophthalmologist involved in managing outsourcing (online)	19/08/2020
#4	Three representatives from service provider 1: medical director, head physician of ophthalmology, deputy chief of ophthalmology	02/09/2020
#5	Three representatives from service provider 2: director of outsourced specialised care, head of ophthalmology, nurse director of ophthalmology	02/09/2020
#6	Representative from service provider 3: head physician of ophthalmology (online)	02/09/2020
#7	Representative from HUS management, head of HUS Head and Neck Center	02/09/2020

### 2.2 Literature reviews

Literature review was done in 2020 from PubMed by each project group member. As the results were so scarce, Google Scholar search was also used to supplement the review. All relevant material of the theoretical background of healthcare outsourcing and used healthcare payment models was uploaded and shared in cloud.

An additional systematic literature review was conducted to explore bundled payment use in ophthalmology in spring 2023. The search, assisted by an information specialist from Helsinki University Library, utilised Elsevier's Scopus and Ovid Medline databases, focusing on English‐language articles. Twenty‐nine different key words were tried in multiple combinations (Table S1) in the search. Despite extensive efforts, only one relevant article was found, covering a report of ophthalmologists' views on bundled payment based on a surveying the members of the New England Ophthalmological Society (NEOS) (Thakore et al., 2016). Additional searches on Google Scholar yielded no further relevant results. Consequently, the planned systematic review was abandoned in favour of a general update on outsourcing methods literature.

### 2.3 SWOT analysis and custom evaluation matrices

SWOT analysis was chosen to facilitate the evaluation of strengths, weaknesses, opportunities and threats related to the models under review. The SWOT analysis is recommended to be used in combination with other qualitative and quantitative techniques (Gürel & Tat, 2017). To accomplish this, three custom‐made matrices were used. The ‘SWOT Matrix’ assessed the strengths, weaknesses, opportunities and threats of each model. Opportunities were evaluated based on external factors that benefit the model and the capability to seizing these opportunities. Similarly, threats were assessed by identifying unfavourable external elements in the environment. The ‘Project Criteria Matrix’ facilitated scoring each model against five criteria for the optimal outsourcing model: timely access to care, quality of care, cost containment, scalability and reduction of administrative workload. The ‘Consolidated Evaluation Matrix’ assessed each option based on three dimensions: desirability, viability and feasibility. Desirability measured the potential to meet the criteria of the optimal model and stakeholder needs, incorporating insights from the Project Criteria Matrix. Viability evaluated the potential to generate value for HUS Eye Hospital by meeting HUS's strategic objectives, integrating insights from the SWOT analysis. Feasibility was defined as the perceived ease of implementation of each model. All parameters were defined beforehand, and each member of the workgroup individually evaluated and scored the models in the context of HUS Eye Hospital. The scoring scales are seen in the figure legends of each matrices (Tables 2, 3, 4). After each member of the group had valued each aspect of matrices, the results were combined. For each aspect, a mean value was calculated. The total score is the sum of the mean scores of each dimension.

**TABLE 2:** Model‐based average scores of SWOT Matrix.

	Strengths	Weaknesses	Opportunities	Threats	Total score
Fee‐for‐service	1.7	−4.0	1.3	−1.7	−2.7
Capitation	3.3	−3.0	3.3	−4.0	−0.3
Bundled payment	5.0	−2.3	4.7	−2.3	5.0
Insourcing	4.7	−2.3	3.7	−2.3	3.7

*Note*: All parameters were defined beforehand, and each of the six members of the project group individually evaluated and scored them. Weaknesses and threats scaled: −5 (high), −3 (medium) or −1 (low), strengths and opportunities scaled: 1 (low), 3 (medium) or 5 (high). Total score being the sum of the average scores.

**TABLE 3:** Model‐based average scores of Project Criteria Matrix.

	Paperwork	Quality	Access	Scalability	Unit cost	Total score
Fee‐for‐service	1.0	3.3	3.7	3.3	1.0	12.3
Capitation	4.3	1.7	2.7	4.3	4.7	17.7
Bundled payment	3.7	4.0	4.0	4.0	4.3	20.0
Insourcing	4.7	4.7	3.3	1.3	2.0	16.0

*Note*: All parameters were defined beforehand, and each of the six members of the project group individually evaluated and scored them. Each dimension scored: 1, 3 or 5. Total score being the sum of the average scores.

**TABLE 4:** Model‐based average scores of Consolidated Evaluation Matrix.

	Desirability	Viability	Feasibility	Total score
Fee‐for‐service	1.7	1.3	4.7	7.7
Capitation	3.5	2.7	2.3	8.5
Bundled payment	4.0	4.0	3.3	11.3
Insourcing	4.5	3.0	1.3	8.8

*Note*: All parameters were defined beforehand, and each of the six members of the project group individually evaluated and scored them. Each dimension scored: 1, 3 or 5. Total score being the sum of the average scores.

### 2.4 Cost analysis

A cost analysis of the previous outsourcing model was conducted to address one of the main research questions, although cost was not a definitive criterion for the optimal model. This was because HUS is obligated to provide care regardless of cost constraints. Activity‐based cost allocation was used to determine the total cost of a hypothetical insourcing alternative, allowing for comparison of current and future costs of outsourced services and identifying potential cost savings through increased efficiency. The total and patient group‐specific costs of outsourced patients in 2019 were used for calculations. Hypothetical costs of other models were not analysed in the same detail as the defined information was evaluated to be adequate for the integrated model comparison.

### 2.5 Risk analysis

The potential risks related to the set goals for the chosen model, that is timely access to care, quality of care, scalability, ability to contain costs and administrative work reduction, were analysed. The risk analysis was performed using a risk probability scaling table (Figure 5). To assess potential risks if the chosen model would fail to fulfil set goals partially or completely, perspectives from both HUS Eye Hospital and private providers were considered. The likelihood of the risk materialising was appraised considering the available data on the chosen model and current circumstances.

## 3 RESULTS

### 3.1 Results of the stakeholder interviews

HUS Eye Hospital management interviews highlighted concerns about the lack of digital communication tools and challenges arising from multiple and incompatible hospital information systems. These issues led to practical difficulties such as manual document scanning, occasional loss of patient data and hindering quality control of the services. Also, a fear of losing medical professionals from HUS to private providers was raised. Proposed improvements included enhancing patient data processing and streamlining paperwork. No objections were raised to any potential outsourcing model. The optimal model was perceived as patient‐centred, prioritising quality of care, service provision and efficiency. Key measures for success included reducing complaints, minimising professionals' paperwork burden and ensuring reasonable costs.

HUS Eye Hospital employee interviews revealed dissatisfaction with the outsourcing process, particularly regarding bureaucratic complexities in decision‐making, referral handling and quality control. Simplifying the process was deemed essential, with a common desire for a unified hospital information system to streamline operations and enhance quality control and complaint management. HUS Eye Hospital's internal specifications and care guidelines were seen as burdensome. The quality of outsourced care was mostly regarded as satisfactory; criticisms were mainly directed at isolated incidents. A negative perception of the private sector was acknowledged, alongside concerns about ongoing competition for medical professionals.

All interviewed private providers expressed a desire for greater autonomy in patient treatment decisions and voiced no concerns about care quality. There were abundant hopes for process improvement, with a focus on streamlining operations to benefit stakeholders. Clear and consistent information flow, encompassing both paperwork and patient care, was emphasised to enable transparent quality monitoring. Criticisms were directed at the heterogeneity of hospital information systems, hindering effective information sharing. Consolidating toward a common system was seen as a key factor for improvement. The competition for medical professionals was acknowledged as impacting service provision and tender participation, with emphasis on provider costs.

Both HUS employees and private providers shared the need for motivating work and the pay equating to the expected workload. Private providers aimed to simplify processes to increase remuneration for medical professionals' time, while HUS sought simplification to allow employees to focus more on patient care and research instead of administrative work for outsourced services. All stakeholder groups acknowledged satisfactory patient treatment, but views on financial aspects varied. HUS aimed to reduce costs, while the providers avoided the subject or referred to possibilities of minimising price increases by improving processes. A common desire was to unify digital communication between HUS and providers, highlighting the importance of a new integrated hospital information system or at the minimum, the use of systems that intercommunicate.

The need and expectations of the patients were assessed from the patient KPIs including feedback and complaints. Timely access to good quality care, simple procedures and continuous care were seen as essential.

**FIGURE 4:**
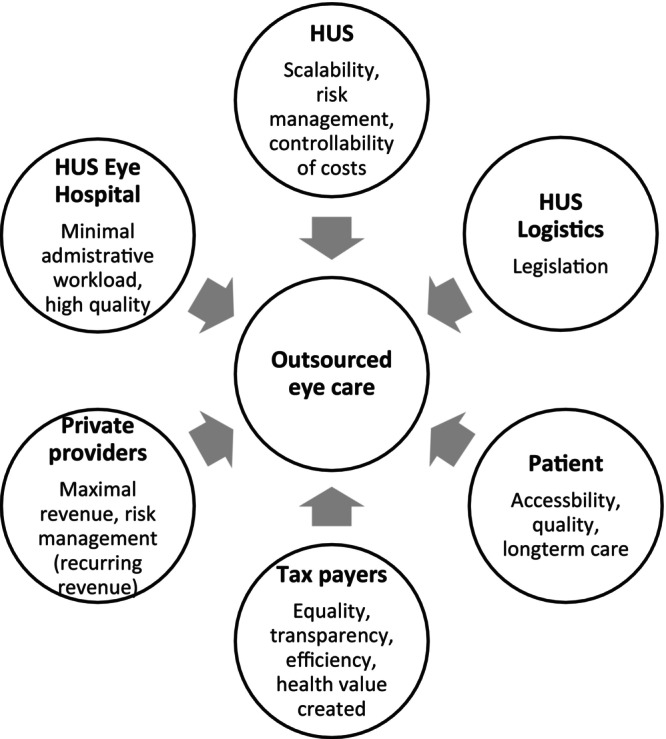
Various stakeholders and their interest in HUS Eye Hospital outsourcing.

The collected data from interviews provided insight into the requirements, expectations and hopes of the different stakeholder groups (Figure 4). Consequently, five criteria were selected as the requirements for the new outsourcing model, namely timely access to care, scalability, quality, ability to contain costs and reduced administrative workload for HUS professionals. All relevant payment models were then evaluated against these criteria.

### 3.2 Results of literature review

Criteria for the optimal solution for HUS Eye Hospital from the stakeholder interviews, best practices from public and private sectors, and from the previous experiences in outsourcing in HUS Eye Hospital guided the literature search.

Some models found in the literature such as service voucher and pay‐for‐performance were discarded on the grounds that their implementation and follow‐up would be impossible or would require unavailable resources under prevailing conditions. Taking these points into consideration, the literature review concentrated on three main outsourcing models: fee‐for‐service, capitation and bundled payment.

In the fee‐for‐service model, the provider is being paid for each visit, procedure, test or other service provided. It suits multiple purposes, as the content of outsourced services can be flexibly modified. It is also useful when temporarily adapting to increasing service volumes. As the provider is being paid by each service, it poses a risk of providing more services than needed, thus incentivising the overutilisation of services (Gosden et al., 2000). This may result in increasing healthcare costs. It may also involve risks in the quality of care, mainly undertreatment, since the content of the visit is left to the discretion of the provider. Fee‐for‐service rewards the quantity but not the quality or efficiency of medical care.

In the capitation model, a provider is contracted to care for a predefined population over a set period, receiving a fixed payment regardless of actual care needs. This fee covers all potential healthcare services, with the provider assuming all related costs and risks. To mitigate financial risks, a ‘stop‐loss mechanism’ can be planned to compensate providers for unforeseen excessive costs. The upside for the provider is that all potential cost savings allow more surplus from the capitation fee to the provider (James & Poulsen, 2016). This model incentivises cost‐saving innovations by the provider by innovating efficient processes and refraining from unnecessary tests and treatments. The downside is the difficulty of adjusting fees for patient risk factors and ensuring the quality of care. Although capitation targets certain costs effectively, it may not fundamentally change healthcare delivery or hold providers accountable for efficiency and outcomes, and it risks limiting patient choice and stifling provider competition (Porter & Kaplan, 2016). In the long term, capitation carries an incentive for the prevention of diseases, as the prevention of a disease usually is less costly than treating it. In short‐term capitation contracts, this is less evident (Gosden et al., 2000).

In bundled payment model, the provider earns a fixed payment for the whole care cycle of a predefined medical condition or episode of care (Porter & Kaplan, 2016). Thus, the bundled payments enable incorporating disease‐specific risk adjustment, which is often more accurate than the population‐based risk adjustment in the capitation model. Episode‐based bundles are used in clearly defined care cycles such as joint replacement or pregnancy and delivery, and time‐based bundles are applied in bundling the care of chronic conditions, such as diabetes or renal diseases (Struijs et al., 2020). Like in the capitation model, in bundled payment, the provider is responsible for all care‐related costs and consequently also gains the benefits from potential cost savings. Thus, it carries a financial risk for providers if treatment costs surpass the set reimbursement (Mechanic & Tompkins, 2012). While it aims to enhance efficiency and reduce costs, concerns exist about potential compromises in care quality (Feder, 2013) and the risk of patient selection as providers may avoid high‐needs or costly patients (Burns, 2013). Also, many patients have multiple chronic conditions and thus it does not provide a comprehensive treatment for the patient (Berenson et al., 2016). However, coupling payments with patient outcomes could mitigate this risk, promoting a value‐based approach that ensures payment aligns with actual value provided.

### 3.3 Results of SWOT analysis and custom‐made evaluation matrices

The results of the SWOT analysis of the three models and insourcing are gathered in Table 5 including the concept in brief, strengths and opportunities, weaknesses and threats, along with key features of models and key milestones for implementation.

**TABLE 5:** SWOT analysis of the reviewed models: concepts, strenghts and opportunities, weaknesses and threats, key features, and implementation milestones. (HUS strategic goals: patient and employee experience, collaboration with wellbeing service counties, continuous improvement, and sustainable economy).

Concept	Concept in brief	Strengths and opportunities for HUS Eye Hospital	Weaknesses and threats to HUS Eye Hospital	Key features	Key milestones
Fee‐for‐service	Separate payment for each visit Applicable to any clearly definite procedure	Suits multiple purposes and different patient groups	May incentivise to overutilisation and cost inflation Does not encourage the providers to develop their processes Risk of cost‐quality trade‐off Creates a competing labour market	Flexible pricing for different kinds of procedures Need for quality control	Categorising patient groups Ensuring efficiency, minimising paperwork
Capitation	Service provider responsible for all the health needs of a patient for a fixed time Payment regardless of actual need	Good continuity of care, provider risk associated with excessive need for care Encourages providers to develop their processes Fixed cost for HUS	Challenges in adjusting the payment ‘Cherry picking’, that is provider choosing the most profitable (healthiest) population ‘Upcoding’, that is claiming multimorbid patients to increase the capitation fee Risk of cost‐quality trade‐off	Fixed monthly/quarterly/yearly payments per patient regardless of needed care A risk‐stratified model Need for quality control	Setting the right fee Risk stratification (based on patient characteristics) Defining criteria for referral (the limits when provider is not responsible)
Bundled payment	Outsourcing the whole treatment cycle of a specified disease	Patient treated by the same provider, which receives fixed fees, thus incentivising increased efficiency HUS allocates patients to providers	Challenges in defining the bundles and bundling multimorbid patients Risk of cost‐quality trade‐off ‘Cherry picking’, that is provider choosing the most profitable (healthiest) patients	Fixed monthly/quarterly/yearly payments based on the disease bundle Need for quality control	Defining right fee for each bundle Risk stratification needed but easier than in capitation ‘Stop‐loss criteria’ Defined procedures for concomitant diseases
Insourcing	HUS Eye Hospital provides all specialised eye care for patients in HUS area No outsourcing of patients	No extra paperwork All treatment and data in one place Simple quality control Easy estimation and control of costs Total control of treatment in HUS	Increases dependence on workforce, facilities and fixed costs	Resembles fee‐for‐service Savings will be returned More facilities and personnel needed	HUS strategy HUS top management view Recruitment of new employees

In the SWOT Matrix model, outsourcing models and insourcing were assessed for their strengths, weaknesses, opportunities and threats (Table 2). Bundled payment scored highest in strengths, opportunities and in total. Fee‐for‐service scored the worst in total. Bundled payment's strengths included outsourcing the patient's disease as an entity, leading to reduced administrative workload and bureaucracy, along with clear incentives for cost efficiency. Its main threat was the challenge of determining the appropriate outsourcing fee, requiring accurate risk stratification. Common weaknesses for all models included the need for quality control and heightened competition for the workforce.

The Project Criteria Matrix assessed the strengths of the three outsourcing models and insourcing across five criteria: paperwork/administrative workload, quality, access, scalability and unit cost (Table 3). Bundled payment scored the highest in total, excelling in reducing administrative burden for HUS personnel, promoting cost efficiency and prioritising patient‐centricity. Fee‐for‐service fared poorly, particularly in paperwork and costs, attributed to its cumbersome decision‐making process. The capitation model showed strength in scalability and unit cost, driven by its strong incentive for cost savings, potentially enhancing efficiency and scalability. The insourcing alternative scored highest in quality and paperwork, benefiting from internal quality control and the absence of outsourcing procedures.

The Consolidated Evaluation Matrix assessed the desirability, viability and feasibility of the models (Table 4). The bundled payment model scored high on desirability, suggesting that the model met the needs and interests of stakeholders well. It also scored the highest on viability, reflecting the potential to deliver the most value to HUS. However, feasibility posed a challenge, indicating the need for careful planning and implementation of new processes and policies. In total, the bundled payment model scored the highest. Fee‐for‐service excelled in feasibility due to its adaptability to changing needs. Insourcing emerged as the most desirable model, likely reflecting a preference among public‐sector employees against outsourcing. Nevertheless, it scored lowest in feasibility, highlighting limitations in facilities and the medical professional workforce at HUS Eye Hospital to handle the volume of eye care patients.

### 3.4 Results of the cost analysis

A cost analysis was conducted to assess the current situation and potential costs of insourcing. The analysis involved determining the structure of patient groups, including visits for examinations, appointments with professionals and treatments, based on invoicing records from outsourced services in 2019 (Table S2). Three key elements—workforce, facilities and equipment—were identified, with contributions from various healthcare professional groups factored into calculations. The capacity of each element for different patient groups was determined annually (annual number of patients per element) using process flow‐through data from 2019 at HUS Eye Hospital. The need for novel facilities and workforce was also calculated using these data (Tables S3 and S4). The costs were assigned accordingly. However, the analysis did not include capital costs due to difficulties in determining alternative costs, nor did it incorporate general costs due to challenges in allocating these costs in the case of major patient volume increases. As fixed costs do not increase proportionately with increasing patient volume, calculations including fixed costs would have overestimated the costs of insourcing (Table S5). The hypothetical insourcing would lead to a larger volume of appointments, affecting the allocation of general costs. However, due to time constraints, the current policy of HUS for general cost allocation was used, which did not adjust for this increased volume. It is important to note this limitation when comparing insourcing calculations with outsourcing costs. Despite these limitations, potential cost savings from streamlined processes associated with insourcing were recognised, including reduced transaction documentation and fewer patient complaints due to improved continuity of care. Using the abovementioned elements, a comparison of the estimated costs of insourcing to the current costs of outsourced services was made (Table S6). The estimated total cost of insourcing was found to be significantly lower than the current cost of outsourcing (respectively, EUR 4.1 million and EUR 7.1 million), keeping in mind that additional costs for increased wages were not included in the analysis it being considered very speculative. However, due to the impossibility of arranging adequate facilities and personnel, insourcing remained a hypothetical option. The results of the cost analysis served as the essential basis for planned fees in an upcoming tender.

### 3.5 Summarising the data and choosing the model

The gathered data was summarised (Table 5) and evaluated, with bundled payment appearing most promising for HUS Eye Hospital compared with capitation or fee‐for‐service models. Estimating costs for a single ophthalmological condition over a set period was easier than for a variable mix of conditions. Many of the risks related to capitation applied also to the bundled payment model, including the risk of consolidation of private providers. After evaluation and taking into account that the bundled payment model scored highest across all custom‐made evaluation matrices, demonstrating strength across all domains, the project group chose bundled payment as the basis of HUS Eye Hospital's new outsourcing model.

### 3.6 Risk assessment of bundled payment

**FIGURE 5:**
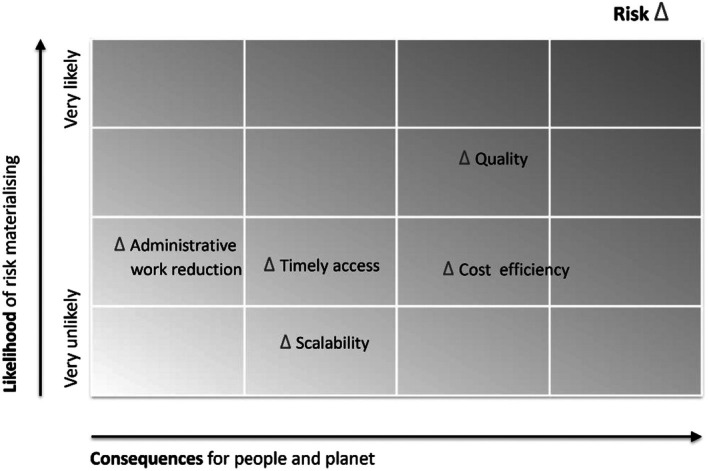
Risk probability scaling assessing the level of impact considering the likelihood and potential consequences if the bundled payment model fails to deliver the expected outcomes. Possible risks were identified from the SWOT analyses, custom‐made evaluation matrices and cost analysis. All parameters were defined beforehand, and each of the six members of the project group individually evaluated and scored them. The likelihood of the risk materialising was appraised considering the available data on bundled payment model and current circumstances.

After selecting the bundled payment model, a risk assessment was conducted to evaluate the likelihood and consequence of not achieving the key outcomes identified from the SWOT analyses, custom‐made evaluation matrices and cost analysis, that is timely access to care, quality of care, scalability, cost containment and reduced administrative workload. The risk analysis utilised a risk probability scaling table (Figure 5) and integrated the findings into the implementation plan and outsourcing tender design to mitigate identified risks. Risks associated with the bundled payment model primarily involved a potential trade‐off between cost and quality of care, with inadequate quality being deemed the highest risk. This highlighted the importance of quality monitoring, leading to the creation of a new common quality register with BCB Medical Ltd., Finland, as a prerequisite for effective quality control. Additionally, challenges in determining the appropriate provider fee level posed a risk of cost inflation yet offered potential savings for HUS Eye Hospital if managed effectively. Monitoring access to care as a part of quality monitoring allows an opportunity to give incentives or sanctions according to results. The scalability of patient volume can be moderated by using several providers and by settling for minimum bundled volumes. The administrative workload should diminish in the transition from fee‐for‐service to the bundled payment model.

### 3.7 The new model

In March 2021, the project group finalised its proposal, creating five disease‐specific bundles (Table 6). Given that retinal disorders, diabetic retinopathy and glaucoma accounted for 90% of outsourcing costs of outpatients, the focus was on these categories. Paediatric and uveitis patients requiring regular follow‐up were also included in bundled payments, while those needing singular visits remained under a fee‐for‐service model with a fixed fee service voucher (SV). Costs from previous fee‐for‐service models and estimated insourcing calculations determined the basis for the bundles fees and service voucher rates. The premise to reduce costs was based on the service providers' possibility to improve the cost efficiency of their processes by allowing more effective resource allocation. Under the bundled payment model, service providers receive reimbursement at predefined intervals after the patient's first appointment, regardless of treatment volume. All treatment required by the bundled disease, except for surgical treatment, remains at the provider's responsibility. Patients needing surgical treatment are referred to HUS Eye Hospital and returned to the provider's care after surgery. Patients with multiple eye diseases receive separate bundles for each condition. If a new ophthalmological symptom arises during the contract period, the provider examines the patient as part of the bundle. If the symptom is related to the ongoing bundle, the treatment and follow‐up continue according to the bundle. If the symptom is due to another ophthalmological disease, the service provider refers the patient to HUS Eye Hospital. HUS Eye Hospital then either opts to treat the disease in‐house or provide the patient with a service voucher or a new bundle for another disease. To avoid the risks of over‐diagnosis, the diagnosis of the diseases in the five bundled disease groups (retina, diabetes, glaucoma, paediatric and uveitis) will be set or confirmed in HUS Eye Hospital before admitting the patient to the private outsourcing provider. The outsourced patients not belonging to the bundled disease groups will require a reasoned referral to be entitled to outsourcing.

**TABLE 6:** SWOT analysis of the rewieved models: concepts, strenghts and opportunities, weaknesses and threats, key features, and implementation milestones. (HUS strategic goals: patient and emplyee experience, collaboration with wellbeing service counties, continious improvement, and sustainable economy).

	Bundles					Fee‐for‐service		
	Retina	Glaucoma	Diabetes	Paediatric	Uveitis	SV1, ophthalmologist visit	SV2, ophthalmologist visit from GP	SV3, examinations
Patient groups/Diagnose	AMD Retinal breaks Retinal degeneration	Suspected glaucoma Diagnosed glaucoma Post‐operative controls	Diabetic retinopathy Retinal vascular diseases	Amblyopia Strabismus	Uveitis Post‐operative controls	Post‐operative controls Nd:YAG laser treatments Eye conditions requiring specialist's checkup	Eye conditions requiring specialist's checkup	OCT Topography Visual field Other

Abbreviations: Nd:YAG, neodymium‐doped yttrium aluminium garnet; OCT, optical coherence tomography.

All accepted private providers must follow the national best practice and HUS Eye Hospitals treatment guidelines, and all medical professionals taking part in the treatment of outsourced patients must be accepted by the head of HUS Eye Hospital. All patients' treatments and examinations will be monitored through the simultaneously launched quality control software (BCB Medical Ltd., Finland). The objective is to evaluate, for example the access to treatment, the number of visits and examinations and main physiological parameters in each bundled entity. Reports from the private providers, the BCB registry and the HUS Eye Hospital's outsourcing management system, Effector (by Polycon Ltd., Finland) will be reviewed by HUS Eye Hospital representatives and each private provider representative twice a year to regularly evaluate the outsourcing service. This and the fixed duration of the outsourcing contracts will hopefully prevent the possible over‐diagnosis of these patients. For all the outsourced patients, a valid referral will be needed for the renewal of the outsourcing contract.

To ensure continuity of care in the event of a change in the private provider, all patient record entries are stored in the national Patient Data Repository (Kanta Services), allowing all healthcare providers to access the documents. Since HUS owns all imaging studies conducted during the outsourcing, a copy of the relevant image is stored in the HUS image archive (PACS = Picture Archiving and Communication System), from where it can be forwarded to the next treating private provider.

**FIGURE 6:**
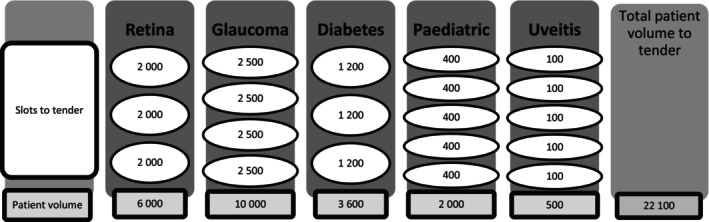
Number and patient volume of the slots in the five disease bundles tendered. Each slot was tendered separately.

Tendering for the new bundled payment outsourcing took place in late 2021. Estimated future patient volumes for the five patient groups were based on previous data and population demographics. Each patient group was subsequently broken down into smaller subgroups to prevent any one group from becoming too large and to allow smaller service providers to take part in the tender. As a result, each of the five patient groups (bundles) was divided into 3–5 competitive subgroups (slots), totalling 20 slots available for tendering (Figure 6). The participating service providers submitted bids for the number of slots they desired within their selected patient groups. Providers could bid for anywhere between 1 and 20 subgroups across the 5 bundles. The outcome of the tendering process was determined by both price and predefined mandatory criteria. If the criteria were met, the lowest price within each patient group (bundle) won the first slot and any additional slots the provider had bid for. Service providers had the option to propose different prices for each subgroup within the same bundle. As a result, the price for each subgroup was determined individually, with the lowest price securing the patients in that particular subgroup. Finally, if a service provider was awarded multiple slots, the average price of their accepted slots was calculated and used as their final bid price. Eventually, the tendered slots were shared among three private providers in the Helsinki area.

Since 1 July 2022, the outpatient outsourcing at HUS Eye Hospital has been running under the new contract, and the BCB quality control software is now in use. Although, its deployment was delayed until 2023 due to compatibility issues with the private provider's hospital information system.

## 4 DISCUSSION

This paper describes the first published bundled payment outsourcing model in eye care. There is a scarcity of literature on the use of alternative reimbursement methods in ophthalmology, with no peer‐reviewed publications in English found during our literature search in 2020 and later in 2023. Given the rising patient volumes, increasing outsourcing and healthcare costs, there is a need to evaluate and develop reimbursement models also suitable for ophthalmology. In response to these challenges, HUS Eye Hospital, Finland's largest publicly funded ophthalmic unit, sought to improve its outsourcing policy. Recognising the limitations of the used fee‐for‐service model, a workgroup conducted a comprehensive analysis, including literature reviews, stakeholder interviews, SWOT analyses, custom‐made evaluation matrices and cost calculations. This evaluation led to the adoption of the bundled payment model, which was estimated to be best suited both financially and from the perspective of stakeholders. Since 1 July 2022, HUS Eye Hospital has been utilising the bundled payment model for its outsourcing and further research will evaluate the impact of the model.

Bundled payment is one reimbursement model supporting the broader goals of value‐based healthcare. It has been especially suitable for well‐defined acute or procedure‐based healthcare of single health problems (Conrad, 2015) such as orthopaedics, particularly on procedures like lower extremity joint replacement surgery (Goude et al., 2021) and hip fracture surgery (Pereira et al., 2023) or cataract surgery (Larsson et al., 2022). It has also been applied to chronic conditions such as renal diseases (Emrani et al., 2023), cardiovascular diseases (Oddleifson et al., 2024) and diabetes management (de Bakker et al., 2012). Review of episode‐based bundled payment models has highlighted their favourable impact on healthcare spending, utilisation and quality (Agency for Healthcare Research and Quality, 2012), particularly in surgical settings, noticing though that the majority of studies were limited to orthopaedic surgery (Hider et al., 2023). However, a long‐term evaluation of bundled payment model for chronic disease management, that is diabetes type 2, chronic obstructive pulmonary disease and vascular risk management patients in the Netherland showed an increase in healthcare expenditure, especially for multimorbidity patients (Karimi et al., 2021). This suggests that payment models for chronic diseases and for patients with multiple chronic disorders require a more comprehensive approach. For that, a Person‐Centred and Integrated Care (PC‐IC) model has already been suggested (Bour et al., 2023).

Regardless of bundled payment being globally used in several specialities, in ophthalmology, only some indications of its use in cataract surgery were found (Hurh et al., 2017; Struijs et al., 2020); however, the results have not been reported. Reasons for this can be manifold. For instance, in Finland, the needs for outsourcing have often been prompt due to, for example changes in the legislation and treatment protocols, and have been executed strictly regionally.

The recommendation from the multiprofessional evaluation group for the new outsourcing model for HUS Eye Hospital was clear. Taking into account the six key success factors in the outsourcing procedure (Foote et al., 2018), the project group, including HUS representatives clinically and financially responsible for the outsourcing, identified the key objectives and tailored the proposal, specified the requirements and also considered the practical feasibilities of the model. The model was designed to promote competition, align objectives and incentives, promote data transparency and ensure HUS Eye Hospital the governance of the project. The study was a comprehensive mixed‐methods multi‐stage analysis utilising quantitative and qualitative analysis. The process involved mapping and understanding stakeholder needs, defining and validating the problem and solution in collaboration with the team and HUS Eye Hospital, assessing HUS Eye Hospital's willingness to wait for the solution, obtaining approval for the proposed solution from HUS management and refining the solution and implementation plans based on feedback.

Striving toward value‐based healthcare, which entails evaluating health outcomes and care quality in relation to costs, is crucial (NEJM Catalyst: What Is Value‐Based Healthcare? 2017). Efforts toward it have also been taken in ophthalmology. However, recent reviews indicate the absence of its comprehensive implementation (Abubakar et al., 2024). To pursue this with our bundled payment model, a new quality control record, that is a structured medical record system BCB Medical Ltd., Finland, was developed concurrently. Its use is mandatory for all private providers taking part in the outpatient outsourcing. This tool, which provides parameters for patient follow‐up and treatment, including visual acuity, the number and frequency of visits and injection treatments, and the changes in visual field examinations, enables HUS Eye Hospital to evaluate potential trade‐offs between cost and quality in the future. Also, adherence to the national best practice and HUS Eye Hospital's treatment guidelines is monitored, along with patient complaints, the patient insurance centre decisions and the Net Promoter Score (NPS) values of service providers. The gathered data will provide insights into the care facilitating necessary adjustments. This is important as continuous revision, including standardising target prices with risk stratification and regional adjustments, is essential for policymakers to continue to uphold the efficacy of value‐based care initiatives as a viable means for care improvement (Baker et al., 2023).

Assessing and mitigating the risks is not a one‐time event, but a repetitive learning process requiring continuous re‐evaluation as the bundled payment model is being implemented. Monitoring of implementation and collecting additional data allow timely execution of necessary adjustments. Following the quality of care for patients with complex and multiple eye diseases will be challenging. Thus, the most complex eye care patients are not included in the outsourcing and are treated in HUS Eye Hospital. This apportionment also prevents the direct comparison of the care in the two groups as in‐house patients tend to have more complex and costly treatment needs. Also, the development of unified quality and outcome metrics to compare in‐house and outsourced patients is hindered by the use of differing and incompatible hospital information systems between each private provider and HUS Eye Hospital. Additionally, HUS's quality control programs must undergo periodic tendering, as required by law.

The weaknesses of the project were partly due to time constraints that prevented testing and piloting the model in real life. As there was no specific referable literature on bundled payment use in ophthalmology or no previous experience with the model, all decisions were made by mirroring the available research information from other specialties and using the expertise of the project group. This favours the possibility of a bias in the comparison of the models in favour of other models than fee‐for‐service, as it being the model with previous experiences.

From the beginning, this project concentrated on outpatients with chronic eye disease that needed continuous, long‐term follow‐up; therefore, the well‐functioning outsourcing of cataract patients was left out. Cataract surgery being a nonrecurring event, its costs and course can be more easily estimated. Including cataract surgery in the reformed outsourcing model could have potentially spurred more private providers to participate in the tendering process and thus reduce the costs via increased competition. As outsourcing of cataract surgery constitutes ca 30% of Hus Eye Hospital's outsourcing costs, it is possible that reforming it may be actual in the future.

Additionally, caution must be exercised when interpreting the calculations for the insourcing alternative because of the allocation of fixed costs and the hypothetical nature of the calculations. The calculations, showing the marked difference in costs favouring insourcing, also raised the question of what if or why not use the capital needed for outsourcing to enhance the operation of HUS Eye Hospital, as the inhousing of outsourced services is the optimal goal of HUS Eye Hospital. Unfortunately, this is not feasible under the current organisation of fixed funding and premises and the imbalance in personnel income opportunities. The legislation obliges HUS EYE Hospital to provide public healthcare and sets strict boundaries for access to treatment, which, with the growing volume of patients, leads to a forced need for outsourcing. Actions to advance the operation of HUS Eye Hospital have been made simultaneously, and this has resulted in an annual increase in services provided by HUS as well (Figure 1).

Changing the outsourcing model is our first attempt to optimise the available resources for eye care, hence publishing future results is important. Our further studies yielding years 2023 and 2024 will report the access to treatment, the number of visits, the impacts of the new model on costs and quality of care, and employee and patient satisfaction. The BCB Medical Ltd., Finland, record also provides us specific clinical metrics for each bundled group for follow‐up. This is mandatory as the risk of undertreatment is ominous. The project group recognised this during the evaluation process as the quality of care was estimated as the highest risk in the bundled payment model (Figure 5).

It should also be noted that this mixed‐method project aimed to identify the most suitable model specifically for HUS Eye Hospital, necessitating adjustments to national requirements and legislation. Healthcare organisations and their financing and legislation vary significantly among countries, making comparisons of outsourcing models challenging. Directly copying successful models from other nations is nearly impossible due to the need for local adaptation. Our model is suited for organisations where healthcare is partly provided by private providers, but it cannot be implemented directly. Also, transitioning to a new model requires time for adjustments, and data gathered during this period may not be valid for final analysis, which also applies to our project. However, we suggest following our evaluation process utilising a multiprofessional team and mixed‐methods analysis in developing and adjusting the reimbursement model for local practices.

In conclusion, the aging population, the growing number of eye patients needing newer, better and more expensive treatments, and the increased visual demands of modern society have led to a growing need for eye care. In Finland, outsourcing is used broadly in ophthalmology as the demand has surpassed the capacity of public eye care in many districts. Along with the growing need for care and outsourcing, the costs have inflated. Recognising this, the use of outsourcing models in ophthalmology calls for research and underscores the importance of ongoing evaluation and adaptation in implementing the models. Our study is a pursuit for this, simultaneously acknowledging the compelling need for further innovative methods and better consumption of resources.

## FUNDING INFORMATION

The work was supported by a grant from the Finnish Ophthalmology Society.

## Supporting information


Tables S1–S6
