# Supplementary material for: Outsourced eye care in the Finnish capital region: Switching from fee‐for‐service to bundled payment model
Source: Acta Ophthalmol. 2025 May 19;104(1):114–29. doi: 10.1111/aos.17523 (PMC12803638; doi:10.1111/aos.17523)
Supplement: Supplementary file 1 — Tables S1–S6 [file AOS-104-114-s001.docx]

**Supportive information**

| Key words |  |  |  |  |  |
| --- | --- | --- | --- | --- | --- |
| #1 bundled payment | #2 capitation | #3 cataract | #4 contracting | #5 contracting out | #6 disease capitation |
| #7 episode-based bundle | #8and#9 externalas(z)ing | #10 eye | #11 eye care | #12 eye disease | #13 eye health |
| #14 eyes | #15 eye service | #16 fee-for-service | #17 fee-per- package | #18 ophthalmic | #19 ophthalmologic |
| #20 ophthalmological | #21 ophthalmology | #22 outsourcing | #23 payment | #24 procurement | #25 reimbursement |
| #26 service coupon | #27 service voucher | #28 subcontracting | #29 time-based bundle |  |  |

**Table S1** Keywords used in literature search in 2023

| \| \| Visit type \| AMD \| Glaucoma \| Diabetes \| Paediatric \| Miscellaneous \| Total \| \| --- \| --- \| --- \| --- \| --- \| --- \| --- \| \| Doctor’s appointments \| 12 000 \| 10 000 \| 7 500 \| 2 000 \| 9 500 \| 41 000 \| \| Nurse’s appointments \| 12 000 \| 10 000 \| 7 500 \| 2 000 \| 9 500 \| 41 000 \| \| Injection visits \| 25 000 \| 0 \| 2 200 \| 0 \| 0 \| 27 200 \| \| Laser Treatments \| 500 \| 500 \| 200 \| 0 \| 0 \| 1 200 \| \| Optometrist visits \| 12 000 \| 10 150 \| 7 500 \| 1 000 \| 8 000 \| 38 650 \| \| OCT visits \| 12 000 \| 12 000 \| 5 000 \| 100 \| 2 375 \| 31 475 \| \| Ultrasound visits \| 100 \| 100 \| 300 \| 0 \| 300 \| 800 \| \| Perimetry visits \| 100 \| 10 000 \| 100 \| 100 \| 500 \| 10 800 \| \| Fundus camera visits \| 4 000 \| 3 000 \| 2 000 \| 200 \| 500 \| 9 700 \| \| Total Patient visits \| 77 700 \| 55 750 \| 32 300 \| 5 400 \| 30 675 \| 201 825 \| \| Patients in average \| 5 312 \| 10 150 \| 3 296 \| 1 851 \| 6 541 \| 27 150 \| \| All visits/patients in average/year \| **14.6** \| **5.5** \| **9.8** \| **2.9** \| **4.7** \| **7.4** \| \| \| --- \| --- \| --- \| --- \| --- \| --- \| --- \| --- \| --- \| --- \| --- \| --- \| --- \| --- \| --- \| --- \| --- \| --- \| --- \| --- \| --- \| --- \| --- \| --- \| --- \| --- \| --- \| --- \| --- \| --- \| --- \| --- \| --- \| --- \| --- \| --- \| --- \| --- \| --- \| --- \| --- \| --- \| --- \| --- \| --- \| --- \| --- \| --- \| --- \| --- \| --- \| --- \| --- \| --- \| --- \| --- \| --- \| --- \| --- \| --- \| --- \| --- \| --- \| --- \| --- \| --- \| --- \| --- \| --- \| --- \| --- \| --- \| --- \| --- \| --- \| --- \| --- \| --- \| --- \| --- \| --- \| --- \| --- \| --- \| --- \| --- \| --- \| --- \| --- \| --- \| --- \| --- \| |
| --- | --- | --- | --- | --- | --- | --- | --- | --- | --- | --- | --- | --- | --- | --- | --- | --- | --- | --- | --- | --- | --- | --- | --- | --- | --- | --- | --- | --- | --- | --- | --- | --- | --- | --- | --- | --- | --- | --- | --- | --- | --- | --- | --- | --- | --- | --- | --- | --- | --- | --- | --- | --- | --- | --- | --- | --- | --- | --- | --- | --- | --- | --- | --- | --- | --- | --- | --- | --- | --- | --- | --- | --- | --- | --- | --- | --- | --- | --- | --- | --- | --- | --- | --- | --- | --- | --- | --- | --- | --- | --- | --- | --- |

**Table S2** Registered approximate customer and visit structure of outsourced patients in 2019. Note that each individual examination and visit has been taken into account separately.

| Estimated capacity of the facilities and workforce as patients/year | | | | | | |
| --- | --- | --- | --- | --- | --- | --- |
|  | **AMD** | **Glaucoma** | **Diabetes** | **Paediatric** | **Miscellaneous** | **Total** |
| Facilities | | | | | | |
| Doctors’ rooms | 2 200 | 2 200 | 1 700 | 2 200 | 2 000 | **10 300** |
| Nurses’ and optometrists’ rooms | 7 400 | 7 400 | 6 900 | 7 400 | 7 200 | 36 300 |
| Injection room | 5 500 | 5 500 | 5 500 | 5 500 | 5 500 | **27 500** |
| Laser rooms | 9 000 | 9 000 | 8 000 | 9 000 | 9 000 | 44 000 |
| Imaging rooms | 23 800 | 25 400 | 24 600 | 23 800 | 24 600 | 122 200 |
| Surgical microscope rooms | 0 | 1 600 | 0 | 0 | 1 600 | 3 200 |
| Total | **47 900** | **51 000** | **46 700** | **47 900** | **49 900** | **243 500** |
|  | | | | | | |
| Workforce | | | | | | |
| Doctors | 2 000 | 2 000 | 1 700 | 2 000 | 2 200 | **9 900** |
| Nurses | 3 000 | 3 000 | 4 000 | 2 000 | 4 000 | **16 000** |
| Nurses (AMD injections) | 5 500 | 5 500 | 5 500 | 5 500 | 5 500 | **27 500** |
| Optometrists | 3 000 | 3 000 | 3 000 | 2 000 | 3 000 | **14 000** |
| Technicians/Photographers | 6 000 | 3 000 | 6 000 | 3 000 | 6 000 | **24 000** |
| Total | **19 500** | **16 500** | **20 200** | **14 500** | **20 700** | **91 400** |

**Table S3** Estimation of facility and workforce capacity per year needed for insourcing based on 2019 customer structure

| Extra capacity needed for estimated insourcing/year | | | | | | |
| --- | --- | --- | --- | --- | --- | --- |
|  | **AMD** | **Glaucoma** | **Diabetes** | **Paediatric** | **Miscellaneous** | **Total** |
| Facilities | | | | | | |
| Doctors’ rooms | 5 | 5 | 4 | 1 | 5 | 20 |
| Nurses’ and optometrists’ rooms | 7 | 7 | 5 | 1 | 7 | 27 |
| Injection rooms | 5 | 0 | 0 | 0 | 0 | 5 |
| Laser rooms | 0 | 0 | 0 | 0 | 0 | 0 |
| Imaging rooms | 3 | 8 | 1 | 0 | 0 | 12 |
| Total | **20** | **20** | **10** | **2** | **12** | **64** |
|  | | | | | | |
| Workforce | | | | | | |
| Doctors | 6 | 5 | 4 | 1 | 4 | 20 |
| Nurses | 4 | 3 | 2 | 1 | 2 | 12 |
| Nurses (AMD injections) | 5 | 0 | 0 | 0 | 0 | 5 |
| Optometrists | 4 | 3 | 3 | 1 | 3 | 14 |
| Technicians/Photographers | 3 | 8 | 1 | 0 | 1 | 13 |
| Total | **22** | **19** | **10** | **3** | **10** | **64** |

**Table S4** Estimated need of extra facility and workforce capacity for insourcing with 2019 customer structure.

| Facility | Total | Furniture | Computer | Equipment | Rent | m2 | €/m2/month | Furniture total | Furniture lifetime | Equipment | Equipment lifetime |
| --- | --- | --- | --- | --- | --- | --- | --- | --- | --- | --- | --- |
| Doctors’ appointment rooms | 17380 | 650 | 2 000 | 2 250 | 12480 | 16 | 65 | 13 000 | 20 | 45 000 | 20 |
| Nurses’ appointment rooms | 13320 | 400 | 2 000 | - | 10920 | 14 | 65 | 8 000 | 20 | - | - |
| Injection rooms | 29900 | 400 | 2 000 | 500 | 27000 | 90 | 25 | 8 000 | 20 | 5 000 | 10 |
| Laser SLT rooms | 17320 | 400 | 2 000 | 4 000 | 10920 | 14 | 65 | 8 000 | 20 | 40 000 | 10 |
| Nd:YAG laser rooms | 16320 | 400 | 2 000 | 3 000 | 10920 | 14 | 65 | 8 000 | 20 | 30 000 | 10 |
| Argon laser rooms | 17320 | 400 | 2 000 | 4 000 | 10920 | 14 | 65 | 8 000 | 20 | 40 000 | 10 |
| Multispot laser rooms | 20320 | 400 | 2 000 | 7 000 | 10920 | 14 | 65 | 8 000 | 20 | 70 000 | 10 |
| Optometrists’ rooms | 17320 | 650 | 2 000 | 2 250 | 12480 | 16 | 65 | 13 000 | 20 | 45 000 | 20 |
| OCT posterior rooms | 22200 | 400 | 2 000 | 12 000 | 7 800 | 10 | 65 | 8 000 | 20 | 120 000 | 10 |
| OCT anterior rooms | 22200 | 400 | 2 000 | 12 000 | 7 800 | 10 | 65 | 8 000 | 20 | 120 000 | 10 |
| Ultrasound rooms | 10950 | 400 | 2 000 | 750 | 7 800 | 10 | 65 | 8 000 | 20 | 15 000 | 20 |
| Perimetry rooms | 16820 | 400 | 2 000 | 3 500 | 10920 | 14 | 65 | 8 000 | 20 | 35 000 | 10 |
| Fundus camera rooms | 15200 | 400 | 2 000 | 5 000 | 7 800 | 10 | 65 | 8 000 | 20 | 50 000 | 10 |
| Surgical microscope rooms | 38000 | 650 | 2 000 | 8 000 | 27000 | 90 | 25 | 20 000 | 20 | 120 000 | 15 |

| Workforce | Average yearly salary (€) including employer’s expenses |
| --- | --- |
| Doctors | 88 000 |
| Nurses | 44 000 |
| Nurses (AMD injections) | 47 000 |
| Optometrists | 49 000 |
| Technicians/photographers | 49 000 |

**Table S5** Breakdown of estimated cost elements for insourcing. (Nd:YAG=Neodymium-doped yttrium aluminium garnet, OCT=Optical coherence tomography, SLT=Selective laser trabeculoplasty)

| Estimate cost of additional insourcing € | | | | | | |
| --- | --- | --- | --- | --- | --- | --- |
|  | **AMD** | **Glaucoma** | **Diabetes** | **Paediatric** | **Uveitis** | **Total** |
| Facilities | | | | | | |
| Doctors’ rooms | 94 800 | 79 000 | 76 676 | 15 800 | 82 555 | 348 831 |
| Nurses’ and optometrists’ rooms | 112 763 | 94 469 | 83 832 | 15 451 | 90 008 | 39 6523 |
| Injection rooms | 135 909 | 0 | 11 960 | 0 | 0 | 147 869 |
| Laser rooms | 3 364 | 3 364 | 2 510 | 0 | 0 | 9 238 |
| Imaging rooms | 62 340 | 128 666 | 24 964 | 1 699 | 14 510 | 232 179 |
| Total | **409 176** | **305 499** | **199 942** | **32 950** | **187 073** | **1 134 640** |
| Workforce | | | | | | |
| Doctors | 528 000 | 440 000 | 388 235 | 88 000 | 380 000 | 1 824 235 |
| Nurses | 176 000 | 146 667 | 82 500 | 44 000 | 104 500 | 553 667 |
| Nurses (AMD injections) | 213 636 | 0 | 18 800 | 0 | 0 | 232 436 |
| Optometrists | 196 000 | 165 783 | 122 500 | 24 500 | 130 667 | 639 450 |
| Technicians/photographers | 132 300 | 409 967 | 60 433 | 6 533 | 30 013 | 639 246 |
| Total | **1 245 936** | **1 162 417** | **6 72 468** | **163 033** | **645 180** | **3 889 034** |
|  | | | | | | |
| Costs total | **1 655 112** | **1 467 916** | **872 410** | **195 983** | **832 253** | **5 023 674** |
|  | | | | | | |
| Estimated workforce savings | | | | | | |
| Less paperwork (3 doctors, 10 secretaries) | -212 174 | -188 177 | -111 837 | -25 124 | -106 689 | -644 001 |
| If place of treatment does not change (2 doctors, 2 secretaries) | -95 544 | -84 738 | -50 361 | -11 131 | -48 043 | -289 817 |
| Total | **-307 718** | **-272 915** | **-162 198** | **-36 255** | **-154 732** | **-933 818** |
|  | | | | | | |
| Total after estimated savings of workforce | 1 347 394 | 1 195 001 | 710 212 | 159 728 | 677 521 | 4 089 856 |
| 2019 paid costs to outsourcing providers | 4 714 901 | 1 412 443 | 512 561 | 155 073 | 336 104 | 7 131 082 |
| Cost difference of estimated insourcing and paid outsourcing in 2019 | **-3 367 507** | **-217 442** | **197 651** | **4 655** | **341 417** | **-3 041 226** |

**Table S6** Estimated costs of hypothetical additional insourcing based on 2019 customer structure and costs compared to 2019 payments of the outsourced services. Result being that insourcing outsourced services would cost ca. 3milj. € less than the outsourcing.
